# Impact of lipodystrophy on health-related quality of life: the QuaLip study

**DOI:** 10.1186/s13023-023-03004-w

**Published:** 2024-01-05

**Authors:** Tevfik Demir, Ilgin Yildirim Simsir, Ozlem Kuman Tuncel, Burcu Ozbaran, Ilker Yildirim, Sebnem Pirildar, Samim Ozen, Baris Akinci

**Affiliations:** 1 Division of Endocrinology and Metabolism, Department of Internal Medicine, Dokuz Eylul University, Izmir, Turkey; 2 Division of Endocrinology and Metabolism, Department of Internal Medicine, Ege University, Izmir, Turkey; 3 Department of Psychiatry, Ege University, Izmir, Turkey; 4 Department of Child and Adolescent Psychiatry, Ege University, Izmir, Turkey; 5 Amryt Pharmaceuticals, Dublin, Ireland; 6 Division of Pediatric Endocrinology and Metabolism, Ege University, Izmir, Turkey; 7 Depark, Dokuz Eylul University, Izmir, Turkey; 8 Izmir Biomedicine and Genome Center, Izmir, Turkey

**Keywords:** Lipodystrophy, Quality of life, Psychiatric disease, Appetite, Emotional well-being

## Abstract

**Background:**

Lipodystrophy is a rare disease characterized by loss of adipose tissue. Natural history studies have demonstrated significant burden of disease; however, there is limited data on the impact of lipodystrophy on quality of life (QoL) and psychoemotional well-being. The QuaLip study is a prospective observational real-world study that aims to determine the impact of lipodystrophy on QoL and psychoemotional well-being and explore subjective burden of the disease. Sixty-seven adult patients and eight pediatric patients with lipodystrophy were included. Patients were followed up for 24 months and assessments were repeated every three months. Patients were examined by licensed psychiatrists at baseline, and at year 1 and year 2 visits.

**Results:**

Eighteen (27.69%) of 65 adult patients (two subjects refused psychiatric assessment) were diagnosed with a psychiatric disorder (e.g., depressive episodes, mixed anxiety and depressive disorder, anxiety disorder, adjustment disorder, recurrent depression, panic disorder, generalized anxiety disorder, unspecified mood disorder, nonorganic sleep disorder, post-traumatic stress disorder, depressive episode comorbidity, social phobia and obsessive–compulsive disorder comorbidity). Lipodystrophy disease and QoL questionnaires revealed a significant disease burden over the study period. More than one-third of patients reported depression symptoms on the Beck Depression Inventory and more than one-fourth of the patients reported significant hunger throughout the study period. Physical appearance, fatigue, and pain contributed to the disease burden. QoL scores were lower in patients with psychiatric disease and in those with poor metabolic control. Attention deficit hyperactivity disorder, depressive disorder, sub-threshold depressive symptoms, obsessive–compulsive disorder, appetite problems, and issues with physical appearance were identified in selected pediatric subjects.

**Conclusions:**

Lipodystrophy has a significant impact on QoL and psychoemotional well-being. Psychiatric disorders seem to be underdiagnosed among patients with lipodystrophy.

**Supplementary Information:**

The online version contains supplementary material available at 10.1186/s13023-023-03004-w.

## Introduction

Lipodystrophy is a rare disease characterized by partial (partial lipodystrophy, PL) or near total loss (generalized lipodystrophy, GL) of adipose tissue. Lipodystrophies are classified as either inherited or acquired, each comprising multiple subtypes [1]. The two main types of inherited lipodystrophies are congenital generalized lipodystrophy (CGL) and familial partial lipodystrophy (FPLD). CGL is characterized by an almost total absence of adipose tissue at birth. In cases of FPLD, body fat distribution is normal at birth, but the loss of fat occurs gradually in a partial fashion, affecting the arms, legs, anterior abdomen, and chest. Several genes responsible for inherited lipodystrophies have been identified, each being associated with different subtypes. Acquired lipodystrophies can develop at any time in life. Underlying autoimmunity can be identified in some patients. While fat loss is generalized in acquired generalized lipodystrophy (AGL), acquired partial lipodystrophy (APL) is characterized by a loss of subcutaneous fat tissue in specific areas, affecting the face, arms, and upper part of the body [2].

Dysfunctional adipose tissue biology in lipodystrophy is associated with leptin deficiency and the dissemination of fat into non-adipose tissues [3]. Deficiency in leptin, a fundamental hormone in energy homeostasis, and ectopic fat accumulation can lead to severe insulin resistance, difficult-to-treat diabetes, and hypertriglyceridemia [4]. These abnormalities often result in end-organ complications (e.g., acute pancreatitis, hepatic cirrhosis, proteinuric renal disease, atherosclerosis) [5, 6].

Natural history studies have highlighted the early onset of severe metabolic complications in patients with lipodystrophy and that these patients often develop end-organ complications at a young age [6–8]. On the other hand, there is a scarcity of data on quality of life (QoL) in patients suffering from non-HIV related lipodystrophy. Although limited, previous studies reported early evidence of poor QoL in lipodystrophy [9–12]. Because lipodystrophy has multiple subtypes and is a heterogeneous disease, this impact can be variable from patient to patient. Hyperphagia is a leading symptom in lipodystrophy which can be more severe in GL due to absolute deficiency of leptin [13]. Physical appearance is one of the main concerns that causes distress and affects QoL [14]. It can be a barrier to social interactions and may lead to social withdrawal, isolation, anxiety, and depression. The psychoemotional impact of lipodystrophy can be a large burden to people living with lipodystrophy. Anxiety and depression are commonly observed [15, 16]. Leptin is an important regulator of gonadal functions. Impaired leptin signaling in lipodystrophy may affect the secretion of gonadotropins and gonadal steroids, which may influence puberty development and fertility [17]. Also, lipodystrophy is associated with polycystic ovary syndrome and hirsutism [1]. These system abnormalities contribute to a high risk of mood problems and emotional issues. Although limited, several previous studies revealed an increased prevalence of mood, anxiety, pain, and eating disorders in lipodystrophy [15, 18, 19].

The QuaLip study is a prospective observational study that is designed to determine the impact of lipodystrophy on QoL, identify psychoemotional symptoms/disorders, explore the patients' experience of lipodystrophy to understand more about the subjective burden of the disease, and observe how this burden changes over time.

## Material and methods

### Study design

This is a naturalistic observational real-world study designed to assess the impact of lipodystrophy on patients’ health outcomes. All patients, who gave consent to participate psychiatrist assessments, were evaluated by licensed psychiatrists at baseline and then annually. The change in the quality of life over time was assessed through standardized health-related quality of life (HRQL) measures. Questionnaires were administered at baseline and then at subsequent time points to understand how outcomes change over time.

### Subjects

Patients with GL and PL, further categorized into congenital and acquired forms, were included in this study. All subjects gave written informed consent and were able to read, understand, and complete survey forms. Participants over the age of 18 were asked to self-complete all the survey forms. In subjects aged between 7 and 18 with a diagnosis of GL or PL (acquired or congenital/familial), consent for inclusion in the study was sought from parents or primary carers of the affected child. Participants sought help from their parents or carers to complete the questionnaires. Patients with a major clinically unrelated comorbidity (e.g., active malignancies, organ dysfunctions caused by any other etiology, psychiatric diseases diagnosed prior to development of lipodystrophy in acquired cases) were excluded.

### Ethical approval

The protocol was reviewed and approved by Ethics Committees (EC) and authorized by the Ministry of Health Turkish Medicines and Medical Devices Agency (MoH TMMDA). The original protocol was approved by the EC on 28.12.2017 and then authorized by the MoH on 17.01.2018.

### Psychiatric assessment

All adult patients were examined by a certified psychiatrist (S.P.) by using a semi-structured psychiatric interview. Psychiatric diagnoses of adult patients were classified according to the mental and behavioral disease criteria of the International Classification of Diseases version 10 (ICD-10). Psychiatric assessments were performed at baseline, year 1, and year 2 visits. Past psychiatric history and family history were evaluated at the initial interview.

A certified child and adolescent psychiatrist (B.O.) completed psychiatric interviews with children and adolescents. Schedule for Affective Disorders and Schizophrenia for School-Aged Children: Present and Lifetime Version (K-SADS-PL) and DSM-5 and DSM-5 criteria were used to ascertain psychiatric diagnoses [20].

### Questionnaires

For adults, patients were asked to fill out the sociodemographic form, lipodystrophy background questions, Short Form-36 (SF36), EQ-5D-5L, Beck Depression Inventory (BDI), Work Productivity and Activity Impairment Questionnaire, Items from the Hyperphagia, Questionnaire for Clinical Trials (HQCT), Items from Derriford Appearance Scale, and Facit Fatigue Scale. For subjects aged 7–17 years, sociodemographic form, lipodystrophy background questions, Peds-QL, EQ-5D-5L, CDI depression test, School Participation Questions, Dykens Hyperphagia Questionnaire, Items from Derriford Appearance Scale (age 13–18 only) were used. Also, patients were asked to answer several nonvalidated lipodystrophy-focused questions.

BDI is a self-report questionnaire with 21 items evaluating the presence and severity of depressive symptoms. Major depression is defined as a score of more than 17 points [21]. SF36 is a generic QoL instrument consisting of 36 questions in eight domains (physical functioning, physical role functioning, bodily pain, social functioning, general mental health, emotional role functioning, vitality, general health perception). Scores for each domain can range between 0 and 100; higher scores indicate better functioning [22]. EQ-5D-5L measures five dimensions of QoL: mobility, self-care, usual activities, pain/discomfort and anxiety/depression [23]. Work Productivity and Activity Impairment Questionnaire is a standard questionnaire for assessing impairments in paid work and activities [24]. HQCT aims to assess the severity of food-seeking preoccupation and behaviours [25]. Derriford Appearance Scale is a validated tool to measure psychological appearance concerns [26]. Facit Fatigue Scale measures the level of fatigue during usual daily activities [27]. The Facit Fatigue total score ranges from 0 to 52. Lower scores indicate greater fatigue. The recall period for each item is the past 7 days. The mean score for the general population was reported as 43 in a previous study [27]. Peds-QL is a standard measure of HRQL in children and adolescents [28]. CDI depression test is a validated multi-perspective assessment of depressive symptoms [29]. School Participation Questions seek information regarding school life. Dykens Hyperphagia Questionnaire is a standard tool to assess hyperphagic behavior [30]. Several of these tools are validated for their use in Turkish.

### Laboratory values and concomitant medications

Laboratory tests and concomitant medications were recorded from medical charts by the investigators. All subjects were leptin naïve at enrollment. Leptin levels were measured at baseline and year 2 visits. To note, per local regulations in Turkey, no data was collected on leptin replacement status during follow-up. It is likely that a subset of subjects with GL received leptin replacement during follow-up which may have caused elevations in leptin levels at the year 2 visit.

### Follow-up

Patient inclusion was completed within 13 months. First patient was enrolled on January 26, 2018. Patients were followed up for 24 months and visits were repeated every three months. The last study visit was completed on April 26, 2021. The overall study duration was 40 months. Participants were preferably left to complete the questionnaires on their own; however, a study site coordinator was available to answer any questions. After the first coronavirus disease 2019 (COVID-19) case was detected in Turkey on March 11, 2020, face-to-face visits were minimized. The site coordinator conducted 88% of the follow-up visits by telephone, and the remaining visits were organized as face-to-face visits with subjects and investigators. However, psychiatric assessments were prioritized, and 90% of the psychiatry visits scheduled during the COVID-19 pandemic were organized as face-to-face visits.

### Data quality assurance

Monitoring procedures were followed to comply with Good Clinical Practice (GCP) guidelines. A site coordinator was assigned to ensure all study-related activities were in place. Direct access to the on-site study documentation and medical records was ensured. Monitoring was conducted by personal visits from a representative of the Clinical Research Organization (CRO) by checking the case report forms for completeness and clarity and by conducting source data verification from the patient records. In addition to the monitoring visits, frequent communications (e-mail, telephone, and messages) by the study monitor to ensure that the investigation was conducted according to protocol design and regulatory requirements.

### Statistical analysis

Given the very low prevalence of lipodystrophy, it was not possible to recruit a large sample of participants. Also, formal sample size estimation was difficult because this study was not designed to test hypotheses or to compare groups. Instead, the study was designed to recruit a representative sample of patients with lipodystrophy and to document how their QoL changes over time. Given these two constraints, we have estimated to include 50 adult and 30 pediatric participants. Although the adult patient target was met during the inclusion process, it was not possible to include the desired number of pediatric patients in the study. As a result, 67 adult participants and eight pediatric participants were included in the study. Therefore, the study analyzes were performed primarily to include the adult population. Pediatric patient data are presented separately. The lowest and highest tertiles of selected metabolic parameters (e.g., triglycerides) were generated to compare measures of QoL in patients with relatively better and worst metabolic control. Continuous parameters are presented as median and 25–75 percentiles due to the skewed distribution. For comparison of SF36 scores with population-based data, mean ± standard deviation (SD) was used. Categorical data are presented as counts and percentages. The Mann–Whitney U test was used to compare two independent groups. Wilcoxon test was utilized to test significant differences between two-time points (i.e., first vs. the last visit). SF36 scores were compared to the population norms [31]. Categorical parameters were compared by using Chi-Square analysis. The change in two-time points (first vs. the last visit) was analyzed with the Mc Nemar test.

## Results

Sixty-seven adult patients and eight pediatric patients were included in the study. The characteristics of the adult study population is presented in Table 1.

**Table 1:** Baseline and year-1 (visit 5) and year-2 (visit 9) visit characteristics of the study cohort

	Baseline (V1) n = 67	Year-1 (V5) n = 61	Year-2 (V9) n = 56
Age (years)	36 (28–50)	41 (32–54)	39 (29.75–51.25)
*Gender*			
Female	54 (80.60%)	48 (78.69%)	46 (82.14%)
Male	13 (19.40%)	13 (21.31%)	10 (17.86%)
*Type of lipodystrophy*			
CGL	16 (23.88%)	14 (22.95%)	13 (23.21%)
APL	10 (14.93%)	8 (13.11%)	9 (16.07%)
FPLD	41 (61.19%)	39 (63.93%)	34 (60.71%)
Family history for lipodystrophy	30 (44.78%)	31 (50.82%)	29 (51.79%)
Diabetes	61 (91.04%)	54 (88.52%)	48 (85.71%)
Antidiabetics	47 (70.15%)	43 (70.49%)	35 (62.50%)
Insulin	40 (59.70%)	35 (57.38%)	27 (48.21%)
Triglyceride lowering drugs	36 (53.73%)	27 (44.26%)	23 (41.07%)
Hepatic steatosis	44 (65.67%)	39 (63.93%)	36 (64.29%)
Hyperphagia	29 (43.28%)	18 (29.51%)	22 (39.29%)
Infertility	6 (11.11%)	9 (14.75%)	6 (10.71%)
Cardiomyopathy	9 (13.43%)	7 (11.48%)	6 (10.71%)
Other cardiac disorders	7 (10.45%)	6 (9.84%)	4 (7.14%)
Pancreatitis	12 (17.91%)	8 (13.11%)	9 (16.07%)
Ophthalmic disease	12 (17.91%)	13 (21.31%)	14 (25.00%)
Renal disease	16 (23.88%)	14 (22.95%)	12 (21.43%)
ESRD requiring dialysis	3 (4.48%)	5 (8.20%)	5 (8.93%)
Autoimmune disorders	8 (11.94%)	6 (9.84%)	6 (10.71%)
Height (cm)	163 (159–168)	162 (158–168)	162 (158–170)
Weight (kg)	60 (52–75)	61 (52–72)	63 (54–73)
BMI (kg/m^2^)	22.82 (20.31–27.12)	22.81 (20.48–26.12)	23.75 (21.11–27.12)
HbA1c (%)	7.2 (5.9–8.4)	7.4 (6.0–8.7)	6.4 (5.7–8.5)
FBG (mg/dL)	126 (93–171)	126 (103–198)	113 (95–174)
Triglyceride (mg/dL)	261 (144–485)	225 (145–444)	197 (137–582)
LDL cholesterol (mg/dL)	110 (92–130)	97 (67–123)	90 (58–121)
HDL cholesterol (mg/dL)	34 (29–45)	36 (31–44)	31 (24–51)
Leptin (ng/mL)	2.91 (0.62–9.77)	NA	3.82 (1.45–10.87)
ALT (U/L)	25 (18–43)	21 (12–30)	27 (18–44)

Data presented as median (25–75 percentiles). Categorical data are shown as n (%)

ESRD, End Stage Renal Disease; BMI, Body Mass Index; HbA1c, Hemoglobin A1c; ALT, Alanine Transaminase

Per local regulations, no data was collected on leptin replacement status during follow-up. All subjects were leptin naïve at baseline; however, it is likely that a subset of subjects with GL received leptin replacement during follow-up that may have caused elevations in leptin levels at year-2 visit

Of the 67 adult patients included in the study, 65 subjects participated in the psychiatrist assessment at baseline (two patients refused psychiatry interviews for personal reasons). Twenty-two patients (33.85%) had a previous history of psychiatric disorder. At the initial visit, 18 of 65 patients (27.69%) were diagnosed with a psychiatric disorder (seven patients with CGL and 11 patients with FPLD). Depressive episodes were diagnosed in five patients. Four subjects had mixed anxiety and depressive disorder. In addition, the following psychiatric diagnoses were made, each in one person: anxiety disorder unspecified, adjustment disorder, recurrent depression, panic disorder, generalized anxiety disorder, unspecified mood disorder, nonorganic sleep disorder, post-traumatic stress disorder, and depressive episode comorbidity, and social phobia and obsessive–compulsive disorder comorbidity. Psychiatric disorders were resolved in two patients at the year 1 visit. However, clinical assessments revealed psychiatric disorders in four additional subjects at the year-1 visit (three patients developed a depressive episode and one patient was diagnosed with social phobia).

Of the 53 patients who attended the year 2 visit, 24 (45.28%) had a diagnosis of psychiatric illness (four (30.77%) patients with CGL, two with APL, and 18 patients with FPLD). Nine of these patients were diagnosed for the first time at this visit. Five patients had a depressive episode, one patient had social phobia, one patient had mixed anxiety and depressive disorder, one patient had adjustment disorder, and one patient had dysthymia diagnoses. All patients diagnosed with a psychiatric disorder were referred to the psychiatry outpatient clinic for treatment.

**Fig. 1:**
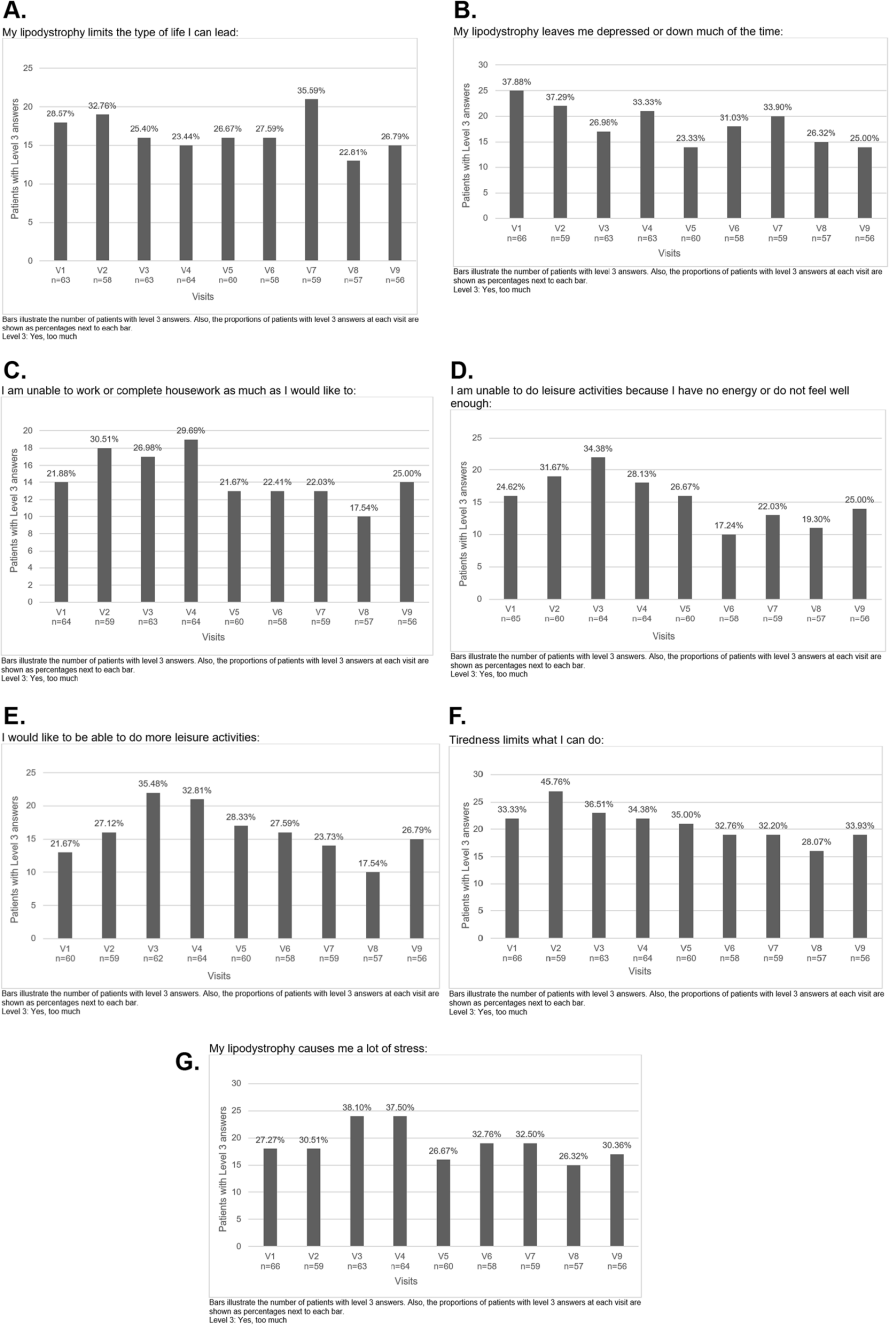
Lipodystrophy disease questionnaires. **A** My lipodystrophy limits the type of life I can lead. **B** My lipodystrophy leaves me depressed or down much of the time. **C** I am unable to work or complete housework as much as I would like to. **D** I am unable to do leisure activities because I have no energy or do not feel well enough. **E** I would like to be able to do more leisure activities. **F** Tiredness limits what I can do. **G** My lipodystrophy causes me a lot of stress

Lipodystrophy disease questionnaires revealed a significant disease burden over the study period. Although slight variations were observed between the visits, generally more than one-fourth of patients found that lipodystrophy limits too much the type of life they can lead (Fig. 1A), leaving them depressed or down much of the time (Fig. 1B), unable to work or complete housework as much as they would like to (Fig. 1C), being unable to do leisure activities because they have no energy or do not feel well enough (Fig. 1D), expressing a wish to be able to do more leisure activities (Fig. 1E), tiredness that limits what they can do (Fig. 1F), and causing them a lot of stress (Fig. 1G).

**Fig. 2:**
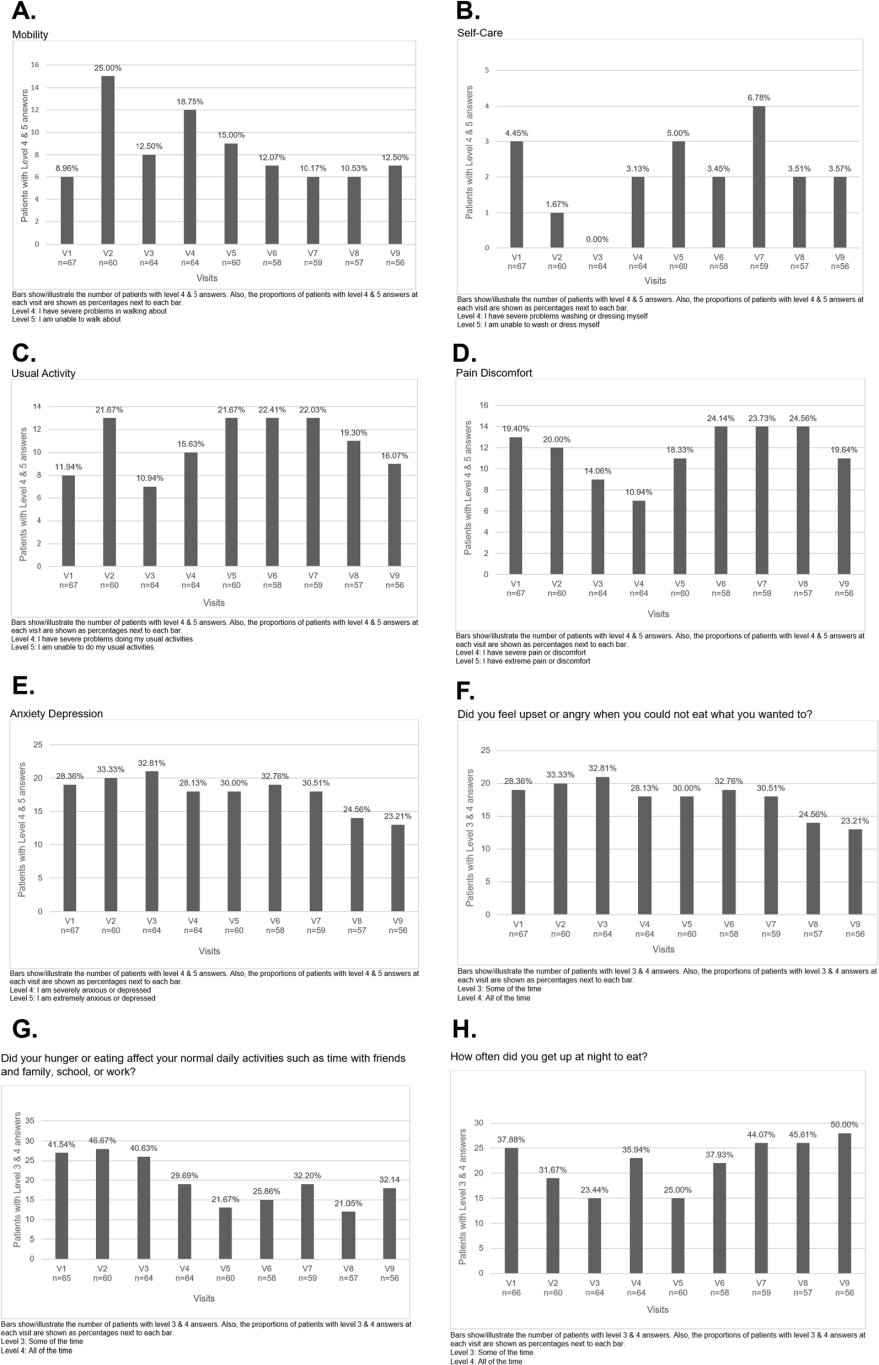
EQ-5D-5L components. **A** Mobility. **B** Self-care. **C** Usual activity. **D** Pain/ discomfort. **E** Depression/anxiety. **F** Did you feel upset or angry when you could not eat what you wanted to? **G** Did your hunger or eating affect your normal daily activities such as time with friends and family, school, or work? **H** How often did you get up at night to eat?

When EQ-5D-5L components were reviewed, a significant proportion of patients reported mobility problems (Fig. 2A), some patients reported self-care issues (Fig. 2B), and a remarkable proportion of patients complained of usual activity (Fig. 2C) issues, pain discomfort (Fig. 2D), and depression/anxiety (Fig. 2E). Over one-third of the adults reported EQ-5D-5L visual analog scale (VAS) scores less than 60 (Table 2), which is associated with poor quality of life. SF36 scores were significantly lower than the Turkish population norms (Table 3). Components of the SF36 scores are presented in Table 3. Generally, females had numerically lower SF36 scores compared to males, though these differences did not reach statistical significance, possibly due to the limited number of males in the study cohort. Changes in SF36 parameters were similar between males and females across the study visits.

**Table 2:** Proportion of patients with EQ-5D-5L VAS score < 60, and Beck Depression Inventory (BDI) and Facit Fatigues scores at each visit

	Baseline (V1) (n = 67)	Month 3 (V2) (n = 60)	Month 6 (V3) (n = 64)	Month 9 (V4) (n = 64)	Year-1 (V5) (n = 60)	Month 15 (V6) (n = 58)	Month 18 (V7) (n = 59)	Month 21 (V8) (n = 57)	Year-2 (V9) (n = 56)
*EQ-5D 5L*									
VAS score < 60, n	20	26	26	25	28	27	26	24	21
VAS score < 60, %	29.85	43.33	40.63	39.06	46.67	46.55	44.07	42.11	37.50
*BDI*									
Median	16.00	17.50	16.50	13.50	12.50	15.50	13.00	17.00	12.50
25 percentiles	7.00	9.00	8.00	06.00	6.00	6.75	5.00	5.00	5.00
75 percentiles	24.00	26.75	26.00	29.25	26.75	29.00	26.00	27.50	25.75
*Facit Fatigues score*									
Median	27.00	25.00	26.50	30.50	32.50	31.50	33.00	33.00	28.00
25 percentiles	17.00	17.25	15.00	16.25	18.00	14.75	17.00	18.50	17.00
75 percentiles	38.00	39.00	40.75	41.00	40.75	41.25	40.00	44.00	45.00

Data presented as median (25–75 percentiles)

BDI, Beck Depression Inventory; VAS, Visual Analogue Scale

Facit Fatigue total score ranges from 0 to 52. Lower scores indicate greater fatigue. Per local regulations, no data was collected on leptin replacement status during follow-up. All subjects were leptin naïve at baseline; however, it is likely that a subset of subjects with GL received leptin replacement during follow-up that may have caused elevations in leptin levels at year-2 visit

**Table 3:** Comparison of Short Form-36 (SF36) scores in patients with lipodystrophy to the population norms of the SF36 Health Survey in Turkey

Population Normal Levels (Men)				SF36 Components in Men with Lipodystrophy at Visit 1				SF36 Components in Men with Lipodystrophy at Visit 9			
SF36	Mean	Std. Deviation	n	Mean	Std. Deviation	n	p (vs. Normal Population)	Mean	Std. Deviation	n	p (vs. Normal Population)
PF	87.2	17.10	609	75.77	20.80	13	0.018	77.27	25.82	11	0.102
RP	89.8	19.30	609	65.38	42.73	13	0.062	79.54	40.02	11	0.198
BP	85.1	16.40	609	60.54	28.50	13	0.006	78.78	40.20	11	0.301
GH	73.6	14.70	609	48.62	23.59	13	0.121	58.63	24.40	11	0.021
VT	65.7	11.90	609	55.77	31.81	13	0.002	76.13	28.75	11	0.889
SF	91.7	12.80	609	70.19	24.23	13	0.008	70.09	27.86	11	0.005
RE	92.8	15.10	609	56.41	39.40	13	0.009	60.00	17.06	11	< 0.001
MH	71.0	10.60	609	63.08	16.42	13	0.283	41.81	31.83	11	0.001

Population Normal Levels (Women)				SF36 Components in Women with Lipodsytrophy at Visit 1				SF36 Components in Women with Lipodsytrophy at Visit 9			
SF-36	Mean	Std. Deviation	n	Mean	Std. Deviation	n	p (vs. Normal Population)	Mean	Std. Deviation	n	p (vs. Normal Population)
PF	80.6	21.70	670	62.13	26.49	54	< 0.001	57.33	30.98	45	< 0.001
RP	82.9	28.60	670	43.19	39.77	54	< 0.001	51.11	48.25	45	< 0.001
BP	81.0	20.20	670	57.65	31.42	54	< 0.001	61.62	28.70	45	< 0.001
GH	69.1	16.90	670	37.63	22.12	54	< 0.001	39.73	24.24	45	< 0.001
VT	63.4	13.70	670	48.06	22.75	54	< 0.001	45.44	25.35	45	< 0.001
SF	90.1	12.90	670	61.56	29.32	54	< 0.001	59.44	30.05	45	< 0.001
RE	89.0	22.50	670	44.00	40.66	54	< 0.001	57.03	47.47	45	< 0.001
MH	70.1	11.40	670	58.04	20.14	54	< 0.001	53.95	22.06	45	< 0.001

Each SF36 scale is directly transformed into a 0–100 scale on the assumption that each question carries equal weight. The lower the score, the more disability. The higher the score, the less disability (a score of zero is equivalent to a maximum disability, and a score of 100 is equivalent to no disability). These scale scores can be combined to produce two component summary scores: the Physical Component Summary (PCS) and the Mental Component Summary (MCS). Data presented as mean and standard deviation

PF, Physical Functioning; RP, Role Limitations due to Physical Health; BP, Pain; GH, General Health; VT, Vitality; SF, Social Functioning; RE, Role Limitations due to Emotional Problems; MH, Mental Health

Per local regulations, no data was collected on leptin replacement status during follow-up. All subjects were leptin naïve at baseline; however, it is likely that a subset of subjects with GL received leptin replacement during follow-up that may have caused elevations in leptin levels at year-2 visit (V9) [31]

**Fig. 3:**
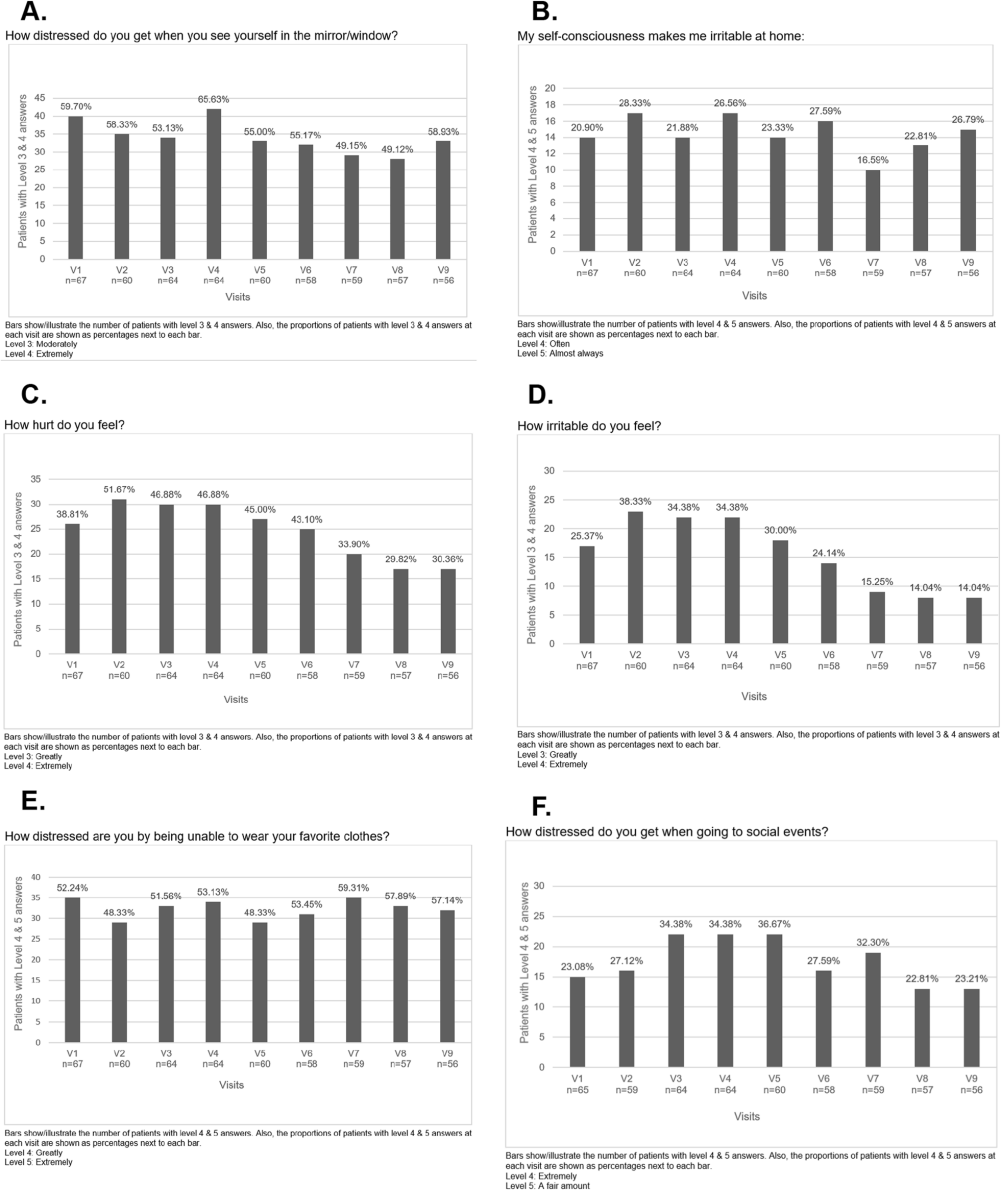
Items from derriford appearance scale. **A** How distressed do you get when you see yourself in the mirror/window? **B** My self-consciousness makes me irritable at home. **C** How hurt do you feel? **D** How irritable do you feel? **E** How distressed are you by being unable to wear your favorite clothes? **F** How distressed do you get when going to social events?

Twenty-five adult patients had Beck Depression Inventory (BDI) scores greater than 17 at the baseline (visit 1, V1) and final (visit 9, V9) visits (37.3% and 44.6%, respectively). BDI scores are presented in Table 2. More than one-fourth of the patients reported significant hunger throughout the study period (Figs. 2F–H). Even though patients with GL exhibited more severe hyperphagia symptoms than those with PL, these differences did not reach statistical significance at baseline (p > 0.05). However, the response to the hyperphagia question "how often did you get up at night to eat?" was significantly influenced (F: 11.204; p = 0.002) by the lipodystrophy type. Patients with GL demonstrated improvements over time, likely attributed to the fact that several GL patients initiated metreleptin therapy after the study commenced. Physical appearance was a significant portion of the disease burden (Fig. 3). Also, patients reported fatigue (Table 2). Only 22 (32.8%) of the adults were employed at baseline.

The impact of the disease on QoL parameters and patient-reported outcomes were comparable in GL and PL although patients with CGL were younger (Table 4). Patients with a psychiatric diagnosis had lower SF36 subscale scores, lower Facit Fatigue scores, and higher BDI scores than those without a psychiatric disorder (Table 5). To assess the effect of metabolic parameters, we created tertiles of baseline triglycerides. Patients in the highest triglyceride tertile had lower weight, BMI, HDL cholesterol, and leptin levels, and higher fasting glucose and HbA1c compared to those in the lowest tertile. SF36 scores were numerically lower in patients with the highest triglyceride tertile; however, the difference between the highest and lowest tertiles were not statistically significant except SF36_RP (Table 6).

**Table 4:** Comparison of patients with GL and PL

	Baseline (V1) (n = 67)			Year-2 (V9) (n = 56)		
	GL (n = 16) (n = 16)	PL (n = 51) (n = 51)	p value	GL (n = 13) (n = 13)	PL (n = 43) (n = 43)	p value
Age (years)	25 (23–30)	42 (32–53)	< 0.001	27 (24–30)	44 (35–55)	< 0.001
Weight (kg)	52 (51–68)	60 (53–71)	0.180	57 (50–72)	66 (56–73)	0.177
BMI (kg/m2)	20.31 (19.45–22.63)	23.62 (20.57–27.12)	0.022	21.04 (18.37–24.56)	24.17 (22.06–27.74)	0.021
Family history	9 (60.0%)	23 (53.5%)	0.662	NA	NA	NA
Diabetes	15 (93.8%)	46 (90.2%)	1.000	11 (84.6%)	37 (86.0%)	1.000
Hypertriglyceridemia	12 (75.0%)	30 (75.0)	1.000	6 (66.7%)	14 (70.0%)	1.000
Antidiabetics	8 (50.0%)	30 (58.8%)	0.534	6 (37.5%)	30 (58.8%)	0.136
Insulin	12 (75.0%)	24 (47.1%)	0.051	6 (37.5%)	22 (43.1%)	0.690
Triglyceride lowering drugs	11 (68.8%)	18 (35.3%)	0.018	6 (37.5%)	17 (33.3%)	0.759
HbA1c (%)	8.3 (6.2–9.5)	7.2 (6.0–8.1)	0.120	6.8 (6.2–8.4)	6.3 (5.6–7.9)	0.449
FBG (mg/dL)	138 (87–170)	122 (96–171)	0.847	111 (98–116)	128 (94–190)	0.824
Triglyceride (mg/dL)	317 (187–457)	222 (155–465)	0.537	475 (144–594)	195 (132–398)	0.532
LDL (mg/dL)	93 (70–101)	116 (95–131)	0.047	73 (57–101)	93 (77–130)	0.180
HDL (mg/dL)	28 (24–35)	37 (32–46)	0.007	30 (24–68)	33 (27–50)	0.973
Leptin (ng/dl)	0.16 (0.10–0.61)	4.73 (1.25–11.69)	< 0.001	3.04 (1.12–30.44)	3.89 (1.52–8.73)	0.643
ALT (U/L)	28 (20–44)	27 (18–41)	0.463	20 (12–32)	18 (15–22)	0.638
SF36_PF	80.00 (45.00–80.00)	70.00 (45.00–90.00)	0.504	65.00 (40.00–100.00)	60.00 (35.00–90.00)	0.380
SF36_RP	37.50 (0.00–75.00)	50.00 (0.00–100.00)	0.526	100.00 (0.00–100.00)	100.00 (0.00–100.00)	0.722
SF36_RE	16.67 (0.00–66.67)	66.67 (0.00–100.00)	0.091	100.00 (0.00–100.00)	100.00 (0.00–100.00)	0.703
SF36_VT	30.00 (28.75–70.00)	55.00 (35.00–67.50)	0.404	60.00 (40.00–65.00)	45.00 (30.00–70.00)	0.726
SF36_MH	52.00 (40.00–63.00)	56.00 (41.00–72.00)	0.418	64.00 (40.00–72.00)	56.00 (40.00–68.00)	1.000
SF36_SF	62.50 (43.75–100.00)	75.00 (37.50–87.50)	0.879	75.00 (50.00–100.00)	62.50 (37.50–87.50)	0.524
SF36_BP	51.50 (22.00–79.00)	52.00 (41.00–84.00)	0.499	74.00 (52.00–100.00)	62.00 (36.50–84.00)	0.295
SF36_GH	32.00 (21.00–48.50)	37.00 (23.50–62.00)	0.461	47.00 (25.00–62.00)	35.00 (20.00–57.00)	0.431
Beck Depression Score	17 (10–23)	14 (7–24)	0.503	9 (3–25)	13 (6–26)	0.607
EQ-5D-5L index value	0.68 (0.31–0.80)	0.68 (0.30–0.84)	0.871	0.75 (0.55–0.91)	0.69 (0.29–0.82)	0.326
EQ-5D-5L VAS	70.00 (40.00–76.25)	75.00 (57.50–90.00)	0.156	80.00 (60.00–85.00)	60.00 (45.00–80.00)	0.192
Facit Fatigue Score	26.50 (16.50–29.75)	27.00 (18.00–39.50)	0.373	42.00 (23.00–45.00)	24.00 (17.00–45.00)	0.466

Sample number (n) changes because some patients have some missing responses. Data presented as median (25–75 percentiles). Categorical data are shown as n (%). P values are calculated using the Mann–Whitney U test for continuous variables and the chi-square test for categorical parameters

CGL, Congenital Generalized Lipodystrophy; FPLD, Familial Partial Lipodystrophy; BMI, Body Mass Index; HbA1c, Hemoglobin A1c; FBG, Fasting Blood Glucose; LDL, Low-Density Lipoprotein; HDL, High-Density Lipoprotein; ALT, Alanine Transaminase; WBC, White Blood Cell Count; SF36_PF, Physical Functioning; SF36_RP, Role Limitations due to Physical Health; SF36_BP, Pain; SF36_GH, General Health SF36_VT, Vitality; SF36_SF, Social Functioning; SF36_RE, Role Limitations due to Emotional Problems; SF36_MH, Mental Health; VAS, Visual Analog Scale. Facit Fatigue total score ranges from 0 to 52. Lower scores indicate greater fatigue. Per local regulations, no data was collected on leptin replacement status during follow-up. All subjects were leptin naïve at baseline; however, it is likely that a subset of subjects with GL received leptin replacement during follow-up that may have caused elevations in leptin levels at year-2 visit

**Table 5:** Comparison of QoL measures in patients with and without a psychiatric diagnosis at the baseline visit

	Diagnosed with a psychiatric disease (n = 18)	No psychiatric diagnosis at the baseline visit (n = 47)	p value
Age	34 (28–42)	37 (27–51)	0.304
*Gender*			0.817
Female	15 (83.33%)	38 (80.85%)	
Male	3 (16.67%)	9 (19.15%)	
*Type of lipodystrophy*			0.099
CGL	7 (38.89%)	9 (19.15%)	
FPLD	11 (61.11%)	29 (61.70%)	
APL	0 (0.00%)	9 (19.15%)	
SF36_PF	50.00 (20.00–85.00)	80.00 (45.00–90.00)	0.033
SF36_RP	12.50 (00.00–50.00)	75.00 (00.00–100.00)	0.015
SF36_RE	00.00 (00.00–33.33)	66.67 (00.00–100.00)	0.005
SF36_VT	30.00 (20.00–55.00)	55.00 (35.00–80.00)	0.002
SF36_MH	40.00 (24.00–56.00)	60.00 (48.00–76.00)	0.003
SF36_SF	43.75 (25.00–62.50)	75.00 (62.50–87.50)	0.001
SF36_BP	45.00 (32.50–57.50)	67.50 (45.00–100.00)	0.014
SF36_GH	20.00 (10.00–30.00)	45.00 (30.00–60.00)	< 0.001
Beck Depression Score	21.50 (14.00–33.00)	14.00 (6.00–22.00)	0.009
EQ-5D-5L index value	0.39 (0.17–0.68)	0.72 (0.33–0.88)	0.008
EQ-5D-5L VAS	50.00 (40.00–75.00)	80.00 (60.00–90.00)	0.004
Facit Fatigue Score	20.50 (16.00–28.00)	29.00 (22.00–43.00)	0.012

Clinical psychiatric evaluation was not completed in 2 (FPLD) patients. Data presented as median (25–75 percentiles). Categorical data shown as n (%). P values are calculated using Mann–Whitney U test for continuous variables and chi square test for categorical parameters

Abbreviations used in the table; CGL, Congenital Generalized Lipodystrophy; FPLD, Familial Partial Lipodystrophy; BMI, Body Mass Index; HbA1c, Hemoglobin A1c; FBG, Fasting Blood Glucose; LDL, Low Density Lipoprotein; HDL, High Density Lipoprotein; ALT, Alanine Transaminase; WBC, White Blood Cell Count; SF36_PF, Physical Functioning; SF36_RP, Role Limitations due to Physical Health; SF36_BP, Pain; SF36_GH, General Health SF36_VT, Vitality; SF36_SF, Social Functioning; SF36_RE, Role Limitations due to Emotional Problems; SF36_MH, Mental Health; VAS, Visual Analog Scale. Facit Fatigue total score ranges from 0 to 52. Lower scores indicate greater fatigue

**Table 6:** Comparison of patients with the lowest and highest baseline triglyceride levels at baseline

	Lowest triglyceride tertile (n = 22)	Highest triglyceride tertile (n = 22)	p value
Age	39 (29–52)	35 (26–44)	0.250
Height (cm)	165 (162–173)	160 (155–165)	0.109
Weight (kg)	67 (57–75)	53 (50–62)	0.009
BMI (kg/m^2^)	24.20 (20.76–28.24)	20.70 (18.37–25.08)	0.033
HbA1c (%)	6.0 (5.6–8.0)	7.3 (6.4–9.3)	0.015
FBG (mg/dL)	95.50 (89.00–145.00)	150.00 (111.00–190.00)	0.022
Triglyceride (mg/dL)	128.50 (100.00–144.00)	608.50 (485.00–888.00)	< 0.001
LDL (mg/dL)	123.50 (95.00–137.00)	113.50 (91.00–125.00)	0.359
HDL (mg/dL)	43.50 (35.00–51.00)	29.50 (25.00–33.50)	0.001
Leptin (ng/mL)	7.06 (2.91–12.77)	0.97 (0.38–4.19)	0.015
ALT (U/L)	23.50 (18–28.50.00)	23.00 (15.00–29.00)	0.876
WBC (10^3^/μL)	7.50 (6.70–8.90)	7.40 (6.80–8.60)	0.900
Hemoglobin (g/dL)	13.00 (12.70–13.80)	12.40 (11.60–13.40)	0.249
Hematocrit (%)	40.00 (37.80–40.70)	36.60 (34.80–39.50)	0.079
Platelet (10^3^/μL)	257.50 (180.50–327.50)	281.00 (221.00–356.00)	0.214
SF36_PF	80.00 (45.00–90.00)	60.00 (45.00–85.00)	0.532
SF36_RP	75.00 (25.00–100.00)	0.00 (0.00–100.00)	0.036
SF36_RE	66.67 (33.33–100.00)	0.17 (0.00–66.67)	0.056
SF36_VT	55.00 (50.00–75.00)	40.00 (20.00–65.00)	0.073
SF36_MH	62.00 (52.00–76.00)	52.00 (40.00–64.00)	0.157
SF36_SF	75.00 (62.50–100.00)	62.50 (37.50–75.00)	0.058
SF36_BP	78.75 (45.00–100.00)	50.00 (32.50–90.00)	0.071
SF36_GH	45.00 (30.00–60.00)	30.00 (30.00–45.00)	0.121
Beck depression score	12.50 (5.00–22.00)	18.00 (7.00–25.00)	0.176
EQ-5D-5L index value	0.71 (0.64–0.88)	0.50 (0.16–0.76)	0.041
EQ-5D-5L VAS	80.00 (75.00–90.00)	67.50 (50.00–80.00)	0.052
Facit fatigue score	32.50 (24.00–42.00)	23.00 (15.00–35.00)	0.069

Data presented as median (25–75 percentiles). Categorical data are shown as n (%). P values are calculated using the Mann–Whitney U test for continuous variables and the chi-square test for categorical parameters

BMI, Body Mass Index; HbA1c, Hemoglobin A1c; FBG, Fasting Blood Glucose; LDL, Low-Density Lipoprotein; HDL, High-Density Lipoprotein; ALT, Alanine Transaminase; WBC, White Blood Cell Count; SF36_PF, Physical Functioning; SF36_RP, Role Limitations due to Physical Health; SF36_BP, Pain; SF36_GH, General Health SF36_VT, Vitality; SF36_SF, Social Functioning; SF36_RE, Role Limitations due to Emotional Problems; SF36_MH, Mental Health; VAS, Visual Analog Scale

All subjects were leptin naïve at baseline. Facit Fatigue total score ranges from 0 to 52. Lower scores indicate greater fatigue

A limited number of pediatric patients (n = 8) were enrolled. Six females and 2 males, aged 8–17 years, were included (3 with CGL, 3 FPLD, and 2 AGL). Characteristics of individual cases are shown in Additional file 1: Table S1. The mean serum leptin level was 2.2 ng/mL, ranging from 0.1 ng/mL to 3.8 ng/mL. All patients had insulin resistance. Three patients had diabetes mellitus, 7 had hepatic steatosis (one with cirrhosis), and 2 had nephropathy. Clinical cirrhosis was also detected in an adult female patient.

Clinical assessments revealed psychiatric problems in several pediatric patients. Attention Deficit Hyperactivity Disorder (ADHD) was present in 4 out of 8 patients whose psychiatric evaluations were completed. A specific learning disorder was present in one of the ADHD patients. In addition, one of the patients with ADHD was diagnosed with depressive disorder at the baseline visit. Sub-threshold depressive symptoms were noted in two patients. One of them had been diagnosed with obsessive–compulsive disorder (OCD). Individual PedsQLC Scores are presented in Additional file 1: Table S2. EQ-5D-5L scores are presented in Additional file 1: Table S3. Dykens Hyperphagia Questionnaire identified appetite problems in selected patients (Additional file 1: Table S4). Similarly, Derriford Appearance Scale revealed issues regarding outer look in a subset of pediatric patients (Additional file 1: Table S5). Out of 8 pediatric patients, 7 were able to continue to school or nursery. This number dropped to 4 at the final visit presumably due to COVID-19-related issues.

## Discussion

In a prospective manner, the QuaLip study evaluated QoL and patient report outcomes in lipodystrophy. Our results reveal that patients living with lipodystrophy have poor QoL scores compared to population norms. Both patients with GL and PL reported poor QoL, although patients with GL were younger. The study also highlights the increased prevalence of psychoemotional disturbances among patients with lipodystrophy. Findings from psychiatrist assessments suggest that psychiatric disorders mostly remain underdiagnosed in lipodystrophy unless evaluated carefully. Lipodystrophy-focused questions further highlighted the emotional disease burden throughout the study visits. Uncontrolled hunger, which is known to be mainly driven by leptin deficiency [32], was a frequent finding. Physical appearance was also identified as an important contributor to disease burden in lipodystrophy.

Standard questionnaires can measure the impact of the disease state on several components of quality of life. Previous cross-sectional studies and patient/caregiver surveys have given us initial insights, indicating that lipodystrophy has a negative impact on the quality of life. Dhankhar et al. [9] reported lower EQ-5D scores in patients with lipodystrophy and further interviews with 12 patients with lipodystrophy revealed low SF36 scores and several issues such as inability to perform usual activities of daily living, inability to attend work/school, impaired mobility, altered physical appearance, fatigue, anxiety, and depression. Our results revealed that physical health was severely affected in patients with lipodystrophy. Most patients were found to score lower than the general population average on the SF36 survey. Patients reported lower SF36 scores in physical functioning, role limitations due to physical health, and general health compared to population norms at baseline. Adult women with lipodystrophy had lower physical health scores than the general population at the year-2 visit [31]. VAS, another measure of general health quality, was less than 60 in almost half of the adult cases, indicating a significant impact on QoL. A significant number of adult patients reported feeling some degree of pain and discomfort. Supporting these findings, patients with lipodystrophy have worse pain-related QoL scores than the general population on the SF36. In parallel to our results, a previous study from the United States reported a high frequency of pain perception and pain being a major driver of quality of life and psychoemotional health in lipodystrophy [15].

The QuaLip study found a substantial psychological and emotional burden among people with lipodystrophy. More importantly, our study, for the first time, reports systematic psychiatric assessments of a cohort of patients with lipodystrophy. All patients were evaluated by licensed psychiatrists. Psychiatric status and emotional well-being were retested annually, revealing the substantial impact of lipodystrophy on psychoemotional health. Our study highlights the value of psychiatric assessments in people affected with lipodystrophy. Patients with lipodystrophy often experience anxiety and depression and these disorders seem to remain underdiagnosed. QoL scores are especially worse in patients suffering from psychiatric disorders. Therefore, recognizing these psychiatric diseases and referring patients for treatment is crucial to reduce the burden of the disease and improve QoL. This approach is also essential to prevent the development of further psychiatric morbidity.

Altered physical appearance is an important disease attribute causing psychological distress and low self-esteem as reported by patients in the QuaLip study. Similar to our findings, the previous Lipodystrophy Patient and Caregiver Survey [33] reported that physical appearance was a barrier to developing new relationships, leading to social withdrawal, isolation, and increasing feelings of anxiety and depression. Patients with lipodystrophy experience additional symptoms including fatigue that may affect everyday life activities such as working and attending school.

Our results highlight that hunger symptoms are likely to impact psychological wellbeing. Loss of leptin signaling affects appetite control leading to severe hyperphagia [34], especially in patients with GL [5, 34]. Hyperphagia was reported as a disease-related attribute in 92% of patients and is thought to have a substantial impact on the psychological well-being of lipodystrophy patients [11]. Due to the subjective state of excessive hunger, it is difficult to capture quantitatively, though it has been linked to a feeling of starvation. It seems important to develop clinical tools that can assess the level of hyperphagia in patients with lipodystrophy.

Patients with lipodystrophy often suffer from severe metabolic disease and organ complications [5]. Everyday life is substantially affected due to the impact and burden of lipodystrophy complications. The metabolic disease seems to be a contributor to poor QoL in lipodystrophy. It is known that low leptin levels and poor diabetes control are associated with symptoms of depression [35, 36]. Also, patients with higher triglyceride levels generally reported numerically lower QoL scores than those with lower triglyceride levels in the QuaLip, suggesting that several components of the metabolic disease in lipodystrophy may have a significant impact on measures of quality of life.

The QuaLip study has several limitations. First, we were not able to enroll a sufficient number of pediatric cases. Although individual assessments can bright some insight, our study cannot effectively measure the impact of lipodystrophy on QoL in pediatric age. Second, our cohort was enriched in CGL and FPLD; other subtypes of lipodystrophy were not represented or underrepresented. Subtype-related features and/or specific molecular etiology may contribute to decreased physical health-related quality. However, our sample size is not adequate to evaluate such a relationship. Although the number of patients with psychiatric diagnoses seems to be increasing during the study period, many factors may have played a role in this. In addition, the COVID-19 pandemic that emerged during the study period may have affected the psychiatrist evaluations, QoL assessments, and patient-reported outcomes in the follow-up visits. The COVID-19 pandemic forced the study team to perform several visits virtually which may have affected our year 1 and year 2 assessments. It is expected that patients with a chronic physical disease would be psychologically vulnerable and fragile during a stressor such as a pandemic. Third, although we used standardized questionnaires to assess different aspects of psychoemotional health and QoL, none of these tools were specifically developed for lipodystrophy. As a next step, one of the authors of this manuscript (B.A.) is leading an initiative within the European Lipodystrophy Consortium (ECLip) to develop lipodystrophy-specific tools that can assess disease burden from a patient’s perspective. Finally, we were not able to collect any information regarding the leptin replacement status of the patients per local regulations in Turkey. Although all patients were leptin replacement therapy naïve at baseline, it is likely that a subset of subjects with GL started leptin replacement afterwards.

In conclusion, the QuaLip study demonstrates that lipodystrophy has a significant impact on QoL and psychoemotional wellbeing of patients which seems to be usually neglected or underdiagnosed in routine clinical practice. A holistic clinical approach is essential to address both physical and emotional needs of patients that can reduce disease burden in lipodystrophy. Psychiatric support and treatment may improve QoL in lipodystrophy but requires the recognition of psychiatric diseases and psychoemotional symptoms by a screening algorithm. Although lipodystrophy is not a curable disease, interventions aiming for better metabolic disease, hunger control, and improved physical appearance may have the potential to improve QoL and psychoemotional symptoms.

### Supplementary Information

**Additional file 1: Table S1.** Characteristics of the pediatric patients. **Table S2.** QoL scores in pediatric patients. **Table S3.** EQ-5D-5L assessment at baseline (V1), year 1 (V5), and year 2 (V9) in pediatric subjects. **Table S4.** Dykens Hyperphagia Questionnaire assessment at baseline (V1), year 1 (V5), and year 2 (V9) in pediatric subjects. **Table S5.** Derriford Appearance Scale assessment at baseline (V1), year 1 (V5), and year 2 (V9) in pediatric subjects.

## Data Availability

Some or all datasets generated during and/or analyzed during the current study are not publicly available but are available from the corresponding author on reasonable request.
