# Supplementary material for: Impact of lipodystrophy on health-related quality of life: the QuaLip study
Source: Orphanet J Rare Dis. 2024 Jan 5;19:10. doi: 10.1186/s13023-023-03004-w (PMC10768358; doi:10.1186/s13023-023-03004-w)
Supplement: Supplementary file 1 — Additional file 1: Table S1. Characteristics of the pediatric patients. Table S2. QoL scores in pediatric patients. Table S3. EQ-5D-5L assessment at baseline (V1), year 1 (V5), and year 2 (V9) in pediatric subjects. Table S4. Dykens Hyperphagia Questionnaire assessment at baseline (V1), year 1 (V5), and year 2 (V9) in pediatric subjects. Table S5. Derriford Appearance Scale assessment at baseline (V1), year 1 (V5), and year 2 (V9) in pediatric subjects. [file 13023_2023_3004_MOESM1_ESM.docx]

**Supplemental Tables**

**Supplemental Table 1**: Characteristics of the pediatric patients

| **General** | Subject ID | 301 | 302 | 303 | 304 | 305 | 306 | 307 | 308 |
| --- | --- | --- | --- | --- | --- | --- | --- | --- | --- |
|  | Gender | Female | Female | Male | Female | Male | Female | Female | Female |
|  | Age at diagnosis | 17 | 13 | 6 | 16 | 6 | 11 | 11 | 7 |
|  | Subtype | APL | AGL | CGL | FPLD | CGL | CGL | FPLD | FPLD |
| **Baseline (V1)** | Age | 17 | 16 | 14 | 16 | 10 | 14 | 13 | 7 |
|  | Height (m) | 1.61 | 1.73 | 1.83 | 1.58 | 1.44 | 1.77 | 1.49 | 1.34 |
|  | Weight (kg) | 38.8 | 62.8 | 84.4 | 39 | 37.4 | 67.9 | 47 | 28 |
|  | BMI (kg/m^2^) | 14.97 | 20.98 | 25.20 | 15.62 | 18.04 | 21.67 | 21.17 | 15.59 |
|  | Concomitant medications | I, TLA | I, OAD | I, OAD, TLA | None | None | OAD, TLA | I, OAD, TLA | None |
|  | Leptin (ng/ml) | 0.76 | 7.11 | 0.1 | 3.87 | 0.62 | 0.53 | 2.69 | 2.64 |
| **Year 1**  **(V5)** | Age | 18 | 17 | 15 | 17 | 11 | 15 | 14 | 8 |
|  | Height (m) | 1.61 | 1.75 | 1.85 | 1.60 | 1.50 | 1.76 | 1.51 | 1.41 |
|  | Weight (kg) | 34 | 59 | 87 | 43 | 37.5 | 67.5 | 49.3 | 32.1 |
|  | BMI (kg/m^2^) | 13.12 | 19.27 | 25.42 | 16.80 | 16.67 | 21.79 | 21.62 | 16.15 |
|  | Concomitant medications | I, TLA | I, OAD | I, OAD | None | None | None | I, OAD, TLA | None |
| **Year 2**  **(V9)** | Age | 19 | 18 | 16 | NA | 12 | 16 | 15 | 9 |
|  | Height (m) | 1.59 | 1.75 | 1.85 | NA | 1.54 | 1.77 | 1.52 | 1.42 |
|  | Weight (kg) | 34.9 | 62 | 94 | NA | 39.4 | 67 | 50 | 33 |
|  | BMI (kg/m^2^) | 13.80 | 20.24 | 27.47 | NA | 16.61 | 21.39 | 21.64 | 16.37 |
|  | Concomitant medications | I, TLA | I, OAD | I, OAD | NA | None | OAD, TLA | I, OAD, TLA | None |
|  | Leptin (ng/ml) | 0.17 | 6.84 | 0.1 | NA | 0.18 | 9.81 | 4.12 | 2.27 |

Abbreviations used in the table; AGL: Acquired General Lipodystrophy; APL: Acquired Partial Lipodystrophy; CGL: Congenital Generalized Lipodystrophy; FPLD: Familial Partial Lipodystrophy; BMI: Body Mass Index; I: Insulin; OAD: Oral antidiabetics; TLA: Triglyceride lowering agents.

**Supplemental Table 2:** QoL scores in pediatric patients

1. Individual PedsQLC scores at baseline (V1), year 1 (V5), and year 2 (V9) in pediatric patients (ages 8-12)

|  | 305_V1 | 305_V5 | 307_V1 | 308_V1 | 308_V5 | 308_V9 |
| --- | --- | --- | --- | --- | --- | --- |
| PedsQLC_1 | 100 | 100 | 50 | 100 | 100 | 100 |
| PedsQLC_2 | 100 | 100 | 25 | 100 | 100 | 100 |
| PedsQLC_3 | 100 | 100 | 0 | 100 | 100 | 100 |
| PedsQLC_4 | 100 | 100 | 75 | 100 | 100 | 100 |
| PedsQLC_5 | 100 | 100 | 75 | 100 | 100 | 100 |
| PedsQLC_6 | 100 | 100 | 0 | 100 | 100 | 100 |
| PedsQLC_7 | 75 | 50 | 25 | 100 | 100 | 100 |
| PedsQLC_8 | 100 | 75 | 50 | 100 | 100 | 100 |
| PedsQLC_9 | 100 | 50 | 50 | 75 | 100 | 75 |
| PedsQLC_10 | 100 | 75 | 50 | 50 | 75 | 75 |
| PedsQLC_11 | 100 | 50 | 0 | 50 | 75 | 50 |
| PedsQLC_12 | 0 | 0 | 25 | 25 | 100 | 50 |
| PedsQLC_13 | 75 | 50 | 0 | 50 | 100 | 100 |
| PedsQLC_14 | 50 | 75 | 25 | 50 | 100 | 75 |
| PedsQLC_15 | 75 | 100 | 25 | 100 | 100 | 100 |
| PedsQLC_16 | 100 | 100 | 50 | 100 | 100 | 100 |
| PedsQLC_17 | 100 | 100 | 75 | 100 | 100 | 100 |
| PedsQLC_18 | 100 | 100 | 50 | 100 | 100 | 75 |
| PedsQLC_19 | 25 | 75 | 0 | 100 | 100 | 50 |
| PedsQLC_20 | 75 | 50 | 25 | 50 | 75 | 50 |
| PedsQLC_21 | 100 | 75 | 25 | 75 | 100 | 50 |
| PedsQLC_22 | 100 | 100 | 25 | 100 | 75 | 75 |
| PedsQLC_23 | 75 | 75 | 75 | 100 | 75 | 100 |
| Mean_PedsQLP | 85.87 | 69.57 | 43.48 | 79.35 | 91.30 | 88.04 |
| SD_PedsQLP | 19.69 | 21.26 | 17.22 | 25.73 | 20.79 | 19.76 |

1. Individual PedsQLP scores at baseline (V1), year 1 (V5), and year 2 (V9) in pediatric patients (ages 8-12)

|  | 305_V1 | 305_V5 | 307_V1 | 308_V1 | 308_V5 | 308_V9 |
| --- | --- | --- | --- | --- | --- | --- |
| PedsQLP_1 | 100 | 100 | 50 | 100 | 100 | 100 |
| PedsQLP_2 | 100 | 100 | 50 | 100 | 100 | 100 |
| PedsQLP_3 | 100 | 75 | 25 | 100 | 100 | 100 |
| PedsQLP_4 | 100 | 100 | 25 | 100 | 100 | 100 |
| PedsQLP_5 | 100 | 100 | 50 | 100 | 100 | 100 |
| PedsQLP_6 | 100 | 50 | 25 | 100 | 100 | 100 |
| PedsQLP_7 | 75 | 75 | 25 | 100 | 100 | 100 |
| PedsQLP_8 | 100 | 50 | 50 | 50 | 100 | 100 |
| PedsQLP_9 | 75 | 50 | 25 | 50 | 100 | 100 |
| PedsQLP_10 | 100 | 75 | 25 | 50 | 100 | 100 |
| PedsQLP_11 | 25 | 50 | 25 | 50 | 75 | 75 |
| PedsQLP_12 | 75 | 25 | 50 | 100 | 100 | 50 |
| PedsQLP_13 | 75 | 75 | 25 | 50 | 100 | 100 |
| PedsQLP_14 | 75 | 50 | 50 | 100 | 100 | 100 |
| PedsQLP_15 | 100 | 50 | 50 | 100 | 100 | 100 |
| PedsQLP_16 | 100 | 75 | 75 | 100 | 100 | 100 |
| PedsQLP_17 | 100 | 75 | 50 | 100 | 100 | 100 |
| PedsQLP_18 | 50 | 100 | 75 | 75 | 100 | 100 |
| PedsQLP_19 | 75 | 50 | 25 | 50 | 50 | 50 |
| PedsQLP_20 | 100 | 75 | 50 | 25 | 50 | 50 |
| PedsQLP_21 | 100 | 50 | 50 | 50 | 25 | 50 |
| PedsQLP_22 | 75 | 75 | 50 | 100 | 100 | 75 |
| PedsQLP_23 | 75 | 75 | 75 | 75 | 100 | 75 |
| Mean_PedsQLP | 85.87 | 69.57 | 43.48 | 79.35 | 91.30 | 88.04 |
| SD_PedsQLP | 19.69 | 21.26 | 17.22 | 25.73 | 20.79 | 19.76 |

1. Individual PedsQLA scores at baseline (V1), year 1 (V5), and year 2 (V9) in adolescents (ages 13-18)

|  | 301 | | | 302 | | | 303 | | | 304 | | 305 | 306 | | | 307 | |
| --- | --- | --- | --- | --- | --- | --- | --- | --- | --- | --- | --- | --- | --- | --- | --- | --- | --- |
|  | V1 | V5 | V9 | V1 | V5 | V9 | V1 | V5 | V9 | V1 | V5 | V9 | V1 | V5 | V9 | V5 | V9 |
| PedsQLA_1 | 100 | 0 | 100 | 25 | 75 | 50 | 100 | 100 | 100 | 100 | 100 | 100 | 100 | 100 | 100 | 0 | 25 |
| PedsQLA_2 | 25 | 0 | 75 | 25 | 50 | 50 | 100 | 100 | 100 | 100 | 100 | 100 | 75 | 100 | 100 | 0 | 0 |
| PedsQLA_3 | 50 | 0 | 75 | 50 | 50 | 50 | 100 | 100 | 100 | 100 | 75 | 100 | 100 | 100 | 100 | 0 | 0 |
| PedsQLA_4 | 100 | 0 | 50 | 25 | 50 | 50 | 100 | 100 | 100 | 100 | 100 | 100 | 75 | 100 | 100 | 0 | 25 |
| PedsQLA_5 | 100 | 0 | 100 | 75 | 100 | 100 | 100 | 100 | 100 | 100 | 100 | 100 | 100 | 100 | 100 | 50 | 100 |
| PedsQLA_6 | 25 | 0 | 100 | 75 | 100 | 75 | 100 | 100 | 100 | 100 | 100 | 100 |  | 100 | 100 | 0 | 25 |
| PedsQLA_7 | 100 | 0 | 75 | 0 | 50 | 100 | 50 | 50 | 75 | 100 | 100 | 75 | 100 | 100 | 75 | 0 | 0 |
| PedsQLA_8 | 100 | 0 | 100 | 0 | 75 | 50 | 75 | 75 | 75 | 100 | 100 | 100 | 100 | 100 | 100 | 0 | 25 |
| PedsQLA_9 | 50 | 50 | 100 | 75 | 100 | 50 | 100 | 100 | 100 | 100 | 75 | 50 | 100 | 100 | 100 | 0 | 50 |
| PedsQLA_10 | 0 | 50 | 100 | 25 | 25 | 0 | 75 | 100 | 50 | 100 | 75 | 100 | 50 | 75 | 50 | 0 | 0 |
| PedsQLA_11 | 25 | 75 | 75 | 50 | 25 | 0 | 75 | 0 | 50 | 100 | 100 | 50 | 25 | 50 | 50 | 0 | 25 |
| PedsQLA_12 | 100 | 25 | 50 | 50 | 75 | 75 | 100 | 100 | 50 | 100 | 100 | 25 | 100 | 100 | 75 | 0 | 50 |
| PedsQLA_13 | 0 | 75 | 100 | 75 | 0 | 0 | 0 | 25 | 50 | 100 | 100 | 100 | 25 | 100 | 75 | 0 | 50 |
| PedsQLA_14 | 50 | 100 | 100 | 100 | 50 | 75 | 100 | 100 | 100 | 100 | 100 | 100 | 50 | 100 | 100 | 0 | 25 |
| PedsQLA_15 | 50 | 100 | 100 | 100 | 100 | 75 | 100 | 100 | 100 | 100 | 100 | 100 | 100 | 100 | 100 | 0 | 50 |
| PedsQLA_16 | 0 | 100 | 100 | 100 | 25 | 25 | 100 | 100 | 100 | 100 | 100 | 100 | 100 | 100 | 100 | 50 | 50 |
| PedsQLA_17 | 50 | 50 | 75 | 75 | 50 | 25 | 100 | 100 | 100 | 100 | 100 | 100 | 100 | 100 | 50 | 0 | 50 |
| PedsQLA_18 | 75 | 50 | 100 | 75 | 50 | 25 | 100 | 100 | 100 | 100 | 100 | 100 | 100 | 100 | 75 | 0 | 25 |
| PedsQLA_19 | 100 | 100 | 100 | 50 | 50 | 50 | 50 | 75 | 75 | 100 | 75 | 50 | 100 | 100 | 75 | 0 | 25 |
| PedsQLA_20 | 25 | 75 | 50 | 25 | 25 | 50 | 50 | 50 | 50 | 100 | 100 | 50 | 75 | 75 | 50 | 0 | 0 |
| PedsQLA_21 | 50 | 100 | 100 | 100 | 25 | 25 | 100 | 100 | 100 | 100 | 100 | 100 | 100 | 100 | 100 | 0 | 0 |
| PedsQLA_22 | 75 | 50 | 100 | 75 | 75 | 100 | 75 | 100 | 50 | 100 | 100 | 100 | 100 | 75 | 50 | 0 | 25 |
| PedsQLA_23 | 25 | 25 | 100 | 0 | 25 | 25 | 25 | 50 | 50 | 75 | 100 | 75 | 100 | 75 | 50 | 25 | 25 |
| Mean_PedsQLA | 55.43 | 44.57 | 88.04 | 54.35 | 56.82 | 48.75 | 85.23 | 87.50 | 81.52 | 98.91 | 95.65 | 85.87 | 85.23 | 93.48 | 81.52 | 5.43 | 28.26 |
| SD_PedsQLA | 36.12 | 39.86 | 18.26 | 33.42 | 26.93 | 32.92 | 22.70 | 22.82 | 22.88 | 5.21 | 9.69 | 23.63 | 25.19 | 13.52 | 21.61 | 14.99 | 24.20 |

1. Individual PedsQLAP scores at baseline (V1), year 1 (V5), and year 2 (V9) in adolescents (ages 13-18)

|  | 301 | | | 302 | | | 303 | | | 304 | | 305 | 306 | | | 307 | |
| --- | --- | --- | --- | --- | --- | --- | --- | --- | --- | --- | --- | --- | --- | --- | --- | --- | --- |
|  | V1 | V5 | V9 | V1 | V5 | V9 | V1 | V5 | V9 | V1 | V5 | V9 | V1 | V5 | V9 | V5 | V9 |
| PedsQLAP_1 | 100 | 25 | 100 | 25 | 75 | 75 | 100 | 100 | 50 | 100 | 100 | 100 | 100 | 100 | 100 | 25 | 25 |
| PedsQLAP_2 | 25 | 25 | 75 | 25 | 50 | 50 | 100 | 100 | 25 | 100 | 100 | 100 | 100 | 100 | 100 | 25 | 25 |
| PedsQLAP_3 | 50 | 25 | 75 | 50 | 50 | 50 | 100 | 100 | 25 | 100 | 100 | 100 | 100 | 100 | 100 | 25 | 25 |
| PedsQLAP_4 | 100 | 25 | 75 | 25 | 100 | 75 | 100 | 100 | 50 | 100 | 100 | 100 | 75 | 100 | 100 | 25 | 25 |
| PedsQLAP_5 | 100 | 25 | 100 | 75 | 100 | 100 | 100 | 100 | 100 | 100 | 100 | 100 | 100 | 100 | 100 | 50 | 25 |
| PedsQLAP_6 | 25 | 25 | 100 | 75 | 50 | 25 | 100 | 100 | 25 | 100 | 100 | 100 | 100 | 100 | 100 | 25 | 25 |
| PedsQLAP_7 | 100 | 25 | 75 | 25 | 50 | 100 | 50 | 75 | 50 | 100 | 100 | 75 | 100 | 100 | 100 | 25 | 25 |
| PedsQLAP_8 | 75 | 25 | 100 | 25 | 50 | 25 | 100 | 75 | 25 | 100 | 100 | 100 | 100 | 100 | 100 | 25 | 25 |
| PedsQLAP_9 | 50 | 50 | 100 | 75 | 100 | 75 | 100 | 100 | 50 | 100 | 100 | 75 | 100 | 100 | 100 | 25 | 50 |
| PedsQLAP_10 | 25 | 50 | 100 | 25 | 50 | 25 | 50 | 100 | 50 | 100 | 100 | 100 | 75 | 75 | 50 | 25 | 25 |
| PedsQLAP_11 | 50 | 75 | 75 | 50 | 25 | 25 | 50 | 25 | 25 | 100 | 75 | 75 | 75 | 50 | 50 | 25 | 25 |
| PedsQLAP_12 | 100 | 50 | 50 | 50 | 75 | 25 | 100 | 100 | 75 | 100 | 100 | 25 | 100 | 100 | 75 | 25 | 50 |
| PedsQLAP_13 | 25 | 75 | 100 | 75 | 25 | 25 | 25 | 50 | 50 | 100 | 100 | 75 | 75 | 100 | 50 | 25 | 50 |
| PedsQLAP_14 | 50 | 100 | 100 | 100 | 75 | 75 | 100 | 100 | 50 | 100 | 100 | 75 | 75 | 100 | 100 | 25 | 50 |
| PedsQLAP_15 | 50 | 100 | 100 | 100 | 100 | 75 | 100 | 100 | 100 | 100 | 100 | 100 | 100 | 100 | 100 | 25 | 50 |
| PedsQLAP_16 | 25 | 100 | 100 | 100 | 25 | 25 | 100 | 100 | 75 | 100 | 100 | 100 | 100 | 100 | 100 | 50 | 50 |
| PedsQLAP_17 | 50 | 50 | 75 | 75 | 75 | 25 | 100 | 100 | 100 | 100 | 100 | 100 | 100 | 100 | 100 | 25 | 50 |
| PedsQLAP_18 | 75 | 50 | 100 | 100 | 50 | 50 | 100 | 100 | 100 | 100 | 100 | 100 | 100 | 100 | 100 | 25 | 50 |
| PedsQLAP_19 | 100 | 100 | 100 | 50 | 50 | 50 | 50 | 75 | 75 | 100 | 100 | 25 | 100 | 100 | 100 | 25 | 25 |
| PedsQLAP_20 | 50 | 75 | 75 | 25 | 25 | 50 | 50 | 50 | 50 | 100 | 100 | 75 | 75 | 75 | 25 | 25 | 25 |
| PedsQLAP_21 | 50 | 100 | 100 | 100 | 50 | 50 | 100 | 100 | 100 | 100 | 100 | 100 | 100 | 100 | 50 | 25 | 25 |
| PedsQLAP_22 | 75 | 50 | 100 | 75 | 100 | 100 | 50 | 100 | 75 | 100 | 100 | 100 | 100 | 75 | 50 | 25 | 25 |
| PedsQLAP_23 | 25 | 25 | 100 | 25 | 25 | 50 | 50 | 50 | 75 | 75 | 100 | 75 | 100 | 75 | 50 | 50 | 25 |
| Mean_PedsQLAP | 59.78 | 54.35 | 90.22 | 58.70 | 59.78 | 53.26 | 81.52 | 86.96 | 60.87 | 98.91 | 98.91 | 85.87 | 93.48 | 93.48 | 82.61 | 28.26 | 33.70 |
| SD_PedsQLAP | 28.94 | 29.82 | 14.58 | 29.78 | 26.90 | 26.44 | 26.35 | 22.45 | 27.00 | 5.21 | 5.21 | 22.39 | 11.22 | 13.52 | 25.49 | 8.61 | 12.17 |

**Supplemental Table 3:** EQ-5D-5L assessment at baseline (V1), year 1 (V5), and year 2 (V9) in pediatric subjects

Data presented as n (%). Abbreviations used in the table; CGL: Congenital Generalized Lipodystrophy; PL: Partial Lipodystrophy; FPLD: Familial Partial Lipodystrophy

**Supplemental Table 4:** Dykens Hyperphagia Questionnaire assessment at baseline (V1), year 1 (V5), and year 2 (V9) in pediatric subjects


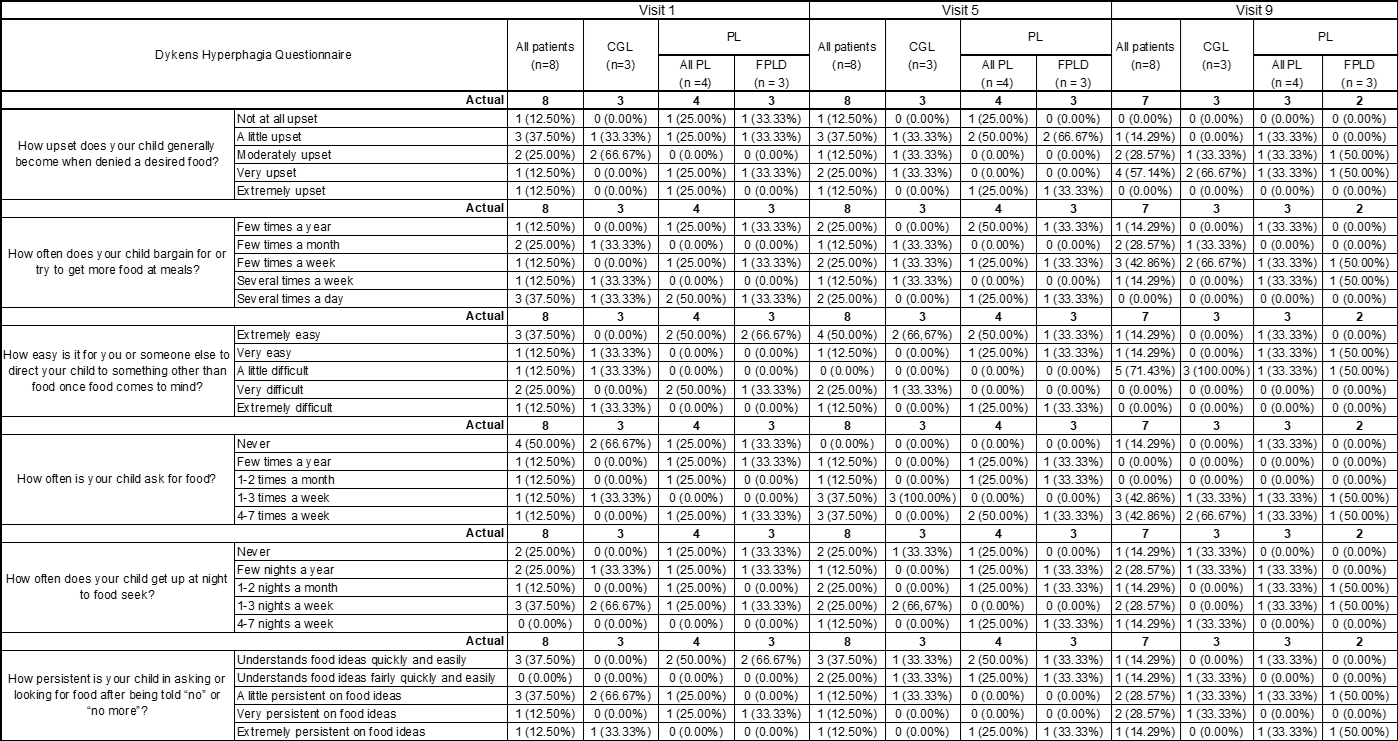

Data presented as n (%). Abbreviations used in the table; CGL: Congenital Generalized Lipodystrophy; PL: Partial Lipodystrophy; FPLD: Familial Partial Lipodystrophy

**Supplemental Table 5:** Derriford Appearance Scale assessment at baseline (V1), year 1 (V5), and year 2 (V9) in pediatric subjects
